# Comparison between cardiovascular magnetic resonance and transthoracic doppler echocardiography for the estimation of effective orifice area in aortic stenosis

**DOI:** 10.1186/1532-429X-13-25

**Published:** 2011-04-28

**Authors:** Julio Garcia, Lyes Kadem, Eric Larose, Marie-Annick Clavel, Philippe Pibarot

**Affiliations:** 1 Québec Heart and Lung Institute, Laval University, Québec, Canada; 2 Laboratory of Cardiovascular Fluid Dynamics, Concordia University, Montréal, Canada

## Abstract

**Background:**

The effective orifice area (EOA) estimated by transthoracic Doppler echocardiography (TTE) via the continuity equation is commonly used to determine the severity of aortic stenosis (AS). However, there are often discrepancies between TTE-derived EOA and invasive indices of stenosis, thus raising uncertainty about actual definite severity. Cardiovascular magnetic resonance (CMR) has emerged as an alternative method for non-invasive estimation of valve EOA. The objective of this study was to assess the concordance between TTE and CMR for the estimation of valve EOA.

**Methods and results:**

31 patients with mild to severe AS (EOA range: 0.72 to 1.73 cm^2^) and seven (7) healthy control subjects with normal transvalvular flow rate underwent TTE and velocity-encoded CMR. Valve EOA was calculated by the continuity equation. CMR revealed that the left ventricular outflow tract (LVOT) cross-section is typically oval and not circular. As a consequence, TTE underestimated the LVOT cross-sectional area (A_LVOT_, 3.84 ± 0.80 cm^2^) compared to CMR (4.78 ± 1.05 cm^2^). On the other hand, TTE overestimated the LVOT velocity-time integral (VTI_LVOT_: 21 ± 4 vs. 15 ± 4 cm). Good concordance was observed between TTE and CMR for estimation of aortic jet VTI (61 ± 22 vs. 57 ± 20 cm). Overall, there was a good correlation and concordance between TTE-derived and CMR-derived EOAs (1.53 ± 0.67 vs. 1.59 ± 0.73 cm^2^, r = 0.92, bias = 0.06 ± 0.29 cm^2^). The intra- and inter- observer variability of TTE-derived EOA was 5 ± 5% and 9 ± 5%, respectively, compared to 2 ± 1% and 7 ± 5% for CMR-derived EOA.

**Conclusion:**

Underestimation of A_LVOT _by TTE is compensated by overestimation of VTI_LVOT_, thereby resulting in a good concordance between TTE and CMR for estimation of aortic valve EOA. CMR was associated with less intra- and inter- observer measurement variability compared to TTE. CMR provides a non-invasive and reliable alternative to Doppler-echocardiography for the quantification of AS severity.

## Background

Accurate assessment of valve stenosis severity is crucial for optimal management of patients with aortic stenosis (AS). The valve effective orifice area (EOA) is one of the most frequently used index to quantify stenosis severity and current ACC/AHA/ESC guidelines propose an EOA < 1.0 cm^2 ^as the criteria to be utilized to identify severe AS [1,2]. Given its non-invasive, radiation-free, low-cost, and versatility nature, transthoracic Doppler-echocardiography (TTE) is currently the method of choice to measure the valve EOA and grade AS severity. However, TTE has several limitations including: i) inability to obtain reliable measurements of EOA due to inadequate acoustic window and poor image quality in some patients; ii) potential for underestimation of flow velocity due to mis-alignment of Doppler beam with flow direction; iii) risk of underestimation of LV outflow (LVOT) diameter due to inadequate quality and/or positioning of image plane; iv) measurement variability related to manual tracing of flow velocity contours, etc [1]. These limitations may significantly alter the performance of TTE to accurately quantify AS severity. Furthermore, the cardiologist if often confronted to discordant results among the different stenotic indices (i.e. EOA, transvalvular gradient, peak velocity, dimensionless velocity index) measured by Doppler-echocardiography or between the Doppler-echocardiographic evaluation of stenosis severity and the patient's clinical status [3]. These discordances may raise some uncertainty about the actual severity of the stenosis and thus about the indication for aortic valve replacement if the patient is symptomatic. When Doppler-echocardiographic evaluation is inconclusive and/or discordant with other clinical findings, catheterization may be used to confirm valve EOA and gradients. However, left heart catheterization is an invasive method that may cause cerebral embolism [4]. Cardiovascular magnetic resonance (CMR) has emerged as a non-invasive, radiation-free alternative modality to corroborate AS severity [5-10]. The majority of previous studies have, however, focused on the evaluation of the valvular anatomic (geometric) orifice area measured by planimetry on the images obtained by CMR or computed tomography [11-13]. From a physiologic standpoint, it is important to emphasize that the transvalvular pressure gradient and thus the LV workload are essentially determined by the valve EOA, i.e. the cross-sectional area of the vena contracta of the transvalvular flow jet, and not by the valve anatomic orifice area [14,15]. And in this regard, it should be noted that the anatomic and effective orifice areas may differ markedly, depending on the magnitude of the flow contraction downstream of the valve.

The objective of this study was to assess the concordance between TTE and CMR for the estimation of valve EOA with use of the continuity equation method.

## Methods

### Study Population

Seven (7) healthy control subjects and 31 patients with mild to severe AS (0.72 cm^2 ^≤ EOA ≤ 1.73 cm^2^) were included in this study. Exclusion criteria were: age < 21 years old, LV ejection fraction < 50%, atrial fibrillation, moderate or severe mitral or aortic regurgitation, poor TTE imaging quality and standard contra-indications to magnetic resonance imaging. All patients provided written informed consent. Initial AS severity classification at study entry was based on TTE-derived EOA: normal (EOA > 2.0 cm^2^), mild (1.5 cm^2 ^< EOA ≤ 2.0 cm^2^), moderate (1.0 cm^2 ^< EOA ≤ 1.5 cm^2^) and severe (EOA ≤ 1.0 cm^2^).

### Transthoracic Echocardiography

TTE studies were performed and analyzed by two experienced echocardiographers. The TTE measurements were performed according to the American Society of Echocardiography guidelines [16] and included: LVOT diameter, LVOT flow velocity by pulsed-wave Doppler, aortic transvalvular jet velocity by continuous-wave Doppler and valve EOA using continuity equation [1]:(1)

Where SV_LVOT _is the stroke volume measured in the LVOT, A_LVOT _is the cross-sectional area of the LVOT calculated assuming a circular shape: (LVOT diameter)^2 ^× 0.785. and VTI_LVOT _VTI_Ao _are the velocity-time integrals of the LVOT and transvalvular flow, respectively.

### Cardiovascular Magnetic Resonance

**Figure 1:**
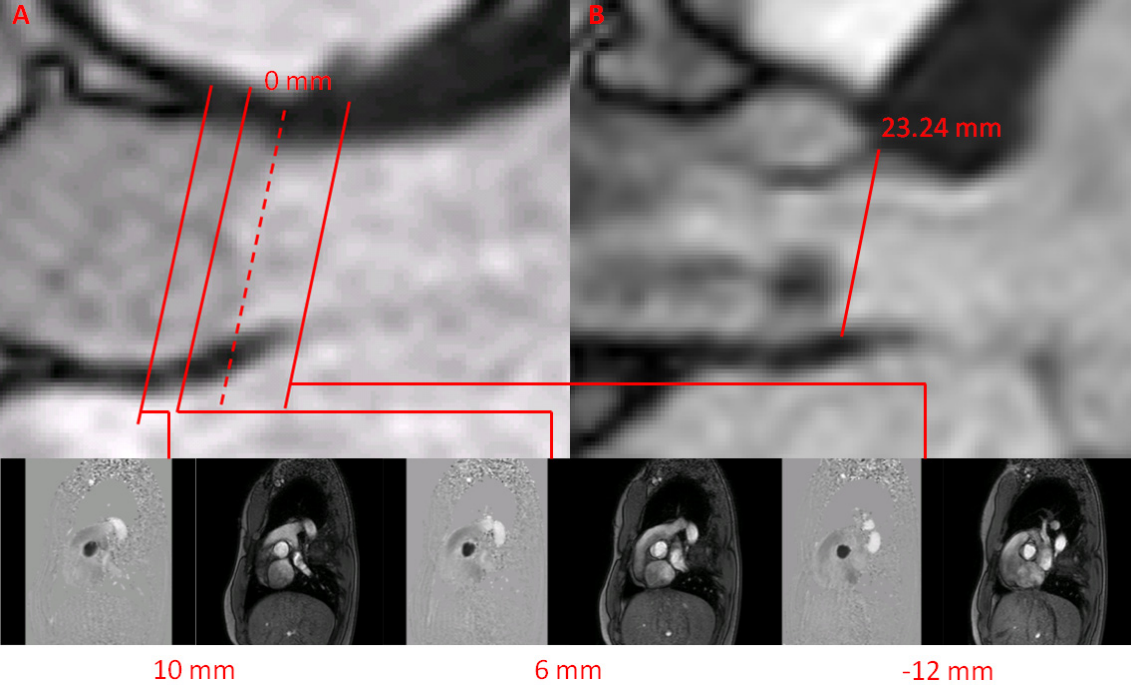
**Image planes used for CMR measurements**. Panel A shows the flow velocity map was acquired at 3 image planes: -12 mm upstream from aortic valve plane (used as the 0 mm reference) and at +6 and +10 mm downstream of the aortic valve plane. The cross-sectional area of the LVOT is measured at the -12 mm position. Panel B shows the measurement of LVOT diameter at annulus location.

CMR studies were performed 2 to 4 weeks after TTE with patients in comparable hemodynamic state. Imaging was performed with a 1.5 Tesla Philips Achieva scanner operating release 2.6 level 3 and dedicated phased-array cardiac coil during successive end-expiratory breath-holds (Philips Healthcare, Best, The Netherlands). Cine imaging of cardiac function was performed by steady-state free precession technique at 30 phases per cardiac cycle (by vectorcardiographic gating) in 8-14 parallel short-axis and 2-chamber, 4-chamber, and 2 orthogonal LVOT planes (8 mm thickness, 0 mm gap). Typical parameters included TR/TE of 3.4/1.2 ms, flip angle 40°, NEX of 1, yielding in-plane spatial resolution of 1.6 × 2 mm. In addition, through-plane phase-contrast (sQFlow SENSE) imaging was performed in the LVOT at 12 mm upstream from the aortic valve annulus (reference: 0 mm) and in the ascending aorta at +6 mm and +10 mm downstream of the annulus (Figure 1). CMR imaging parameters consisted of: TR/TE of 4.60-4.92/2.76-3.05 ms, flip angle 15°, 24 phases, pixel spacing 1.32-2.07 mm, slice thickness 10 mm and acquisition matrix of 256 × 208. Each phase-contrast velocity mapping acquisition produced 2 cine images: one magnitude image and one phase image. For each patient, peak aortic jet velocity measured by TTE was used to define CMR encoding velocity (CMR encoding velocity = (1.25 to 1.5) × peak jet velocity) to optimally define resolution.

**Figure 2:**
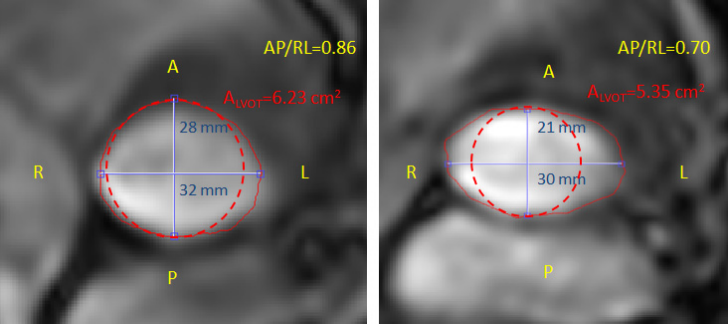
**Left ventricular outflow tract dimensions and cross-section area measurements by CMR**. Left ventricular outflow tract (LVOT) cross-sectional (A_LVOT_; red solid line), anterior-posterior (AP) diameter (blue line), right-left (RL) diameter (blue line), and AP/RL diameter ratio for two different patients. The dashed red line represents the cross-sectional area of LVOT estimated on the basis of the AP diameter and assuming a circular LVOT shape. This estimation yielded values of LVOT cross-sectional area of 6.15 and 3.46 cm^2 ^for these 2 patients compared to the actual area of 6.23 and 5.35 cm^2^, respectively.

CMR images acquisitions and analyses were performed by investigators blinded to clinical and TTE results. A custom-made research application was developed using Matlab software (Mathworks, Natick, Ma) to process and analyze velocity-encoded images [17]. Spatial resolution of CMR images was artificially improved by a factor of three using bicubic averaged interpolation and the magnitude image stack was processed to filter background noise. Regions of interest (ROIs) were defined on each of the 24 phases of magnitude images to include the lumen of the LVOT and of the aorta. The following measurements were performed within each ROI: i) on magnitude images: anterior-posterior (AP) diameter, left-right (LR) diameter, and cross-sectional area of LVOT at the -12 mm position; the ratio of AP/LR diameters was calculated to characterize the shape of LVOT (the lower the ratio the more oval the shape of LVOT) (Figure 2) and ii) on matched phase images: velocity profiles at -12 mm, +6 and +10 mm positions.

**Figure 3:**
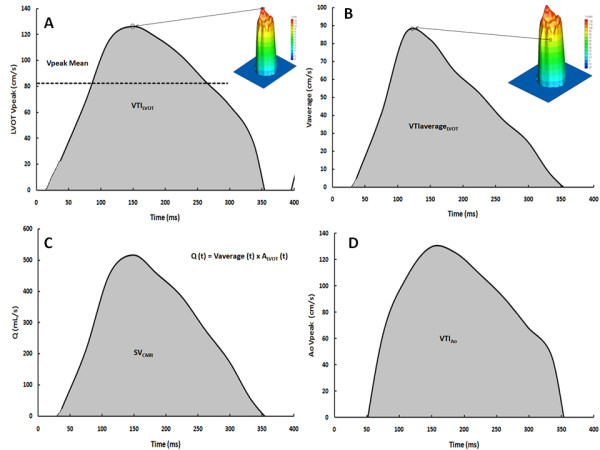
**Flow velocity measurements in the left ventricular outflow tract by CMR**. Panel A shows the change in peak left ventricular outflow tract (LVOT) velocity at -12 mm position during the cardiac cycle. Panel B shows the change in the instantaneous average velocity obtained over the region of interest. The velocity-time integral (VTI) is the area under the curve. Panel C shows the change in instantaneous flow (Q) calculated as follows: Q (t) = average velocity (t) × A_LVOT_, where A_LVOT _is the cross-sectional area of the LVOT. The stroke volume (SV) is the flow-time integral during systole. Panel D shows the change in peak aortic velocity at +6 mm position during the cardiac cycle, the velocity-time integral (VTI) is the area under the curve.

The peak and average flow velocities within the ROI were used to determine the changes in instantaneous peak (V_peak_, Figure 3A) and average (V_average_, Figure 3B) velocity in the LVOT at the -12 mm position during the cardiac cycle. The velocity-time integral of V_average _during systole was calculated (Figure 3B) and compared to the VTI measured by TTE in the LVOT. The instantaneous LVOT flow rate was calculated by multiplying the instantaneous V_average _by the LVOT cross-sectional area, and the stroke volume (SV_CMR_) was calculated by using Simpson's rule to integrate flow during systole (Figure 3C).

The peak flow velocity within the ROI was used to determine the instantaneous peak aortic velocity at the 6 and 10 mm positions (Figure 3D). The velocity-time integral of peak velocity during systole was calculated (VTI_Ao_) and compared to the VTI_Ao _measured by TTE. Given that slightly higher velocities were obtained at 6 mm versus 10 mm, we used the 6 mm position for estimation of VTI_Ao _and EOA by CMR in this study.

The CMR-derived EOA (EOA_CMR_) was then calculated with the following formula:(2)

Where SV_CMR _is the stroke volume using Simpson's rule to integrate systolic flow and VTI_Ao _is the velocity-time integral of the peak aortic flow velocity measured at 6 mm downstream of the valve during systole.

### Measurement variability

To evaluate the intra- and inter- observer variability related to image analysis by CMR and TTE; the measurements of EOA were repeated in a subset of 15 studies (11 AS patients and 4 control subjects) by two blinded observers with the use of the same set of TTE and CMR images. To further evaluate the intra- and inter- observer- variability related to image acquisition and analysis by TTE and CMR, 5 AS patients were imaged twice within 4 weeks (including image acquisition and analysis).

### Statistical analyses

Results are expressed as mean ± SD. CMR versus TTE measurements were compared by 2-tailed paired Student *t*-tests. Correlations and agreements between CMR and TTE measurements were assessed by Pearson's correlations and Bland-Altman comparisons, respectively. Statistical analysis was performed with SPSS 17 (SPSS, Chicago, IL).

## Results

Thirty-one patients with mild to severe AS (77% men, age 67 ± 12 years) and seven healthy subjects (71% men, age 34 ± 8 years) were studied by TTE and CMR. Valve morphology was bicuspid in nine of the 31 AS patients and indeterminate by TTE in 3 patients. Patient characteristics are reported in Table 1.

**Table 1:** Patient Characteristics

Age (years)	62 ± 17
Male gender n (%)	29 (76)
Heart rate (bpm)	65 ± 12
Weight (Kg)	76 ± 13
Height (cm)	169 ± 10
Body surface area (m^2^)	1.88 ± 0.19
Body mass index (Kg/m^2^)	26 ± 3
Valve morphology	
Tricuspid n (%)	26 (68)
Bicuspid n (%)	9 (24)
Indeterminate n (%)	3 (8)

The table shows the mean ± SD or number of patients and percentage.

### LVOT cross-sectional area

LVOT cross-sectional area obtained by TTE was smaller than that obtained by CMR (bias = -0.94 cm^2^, agreement limits: -2.62 to + 0.74 cm^2^) (Table 2). This is, in large part, due to the fact that TTE assumes a circular shape of LVOT and uses the smaller (AP diameter) to compute A_LVOT_, whereas CMR reveals that LVOT shape is oval in the vast majority of patients (Figure 2). The LR and AP LVOT diameters measured by CMR were: 28 ± 3 mm and 24 ± 3 mm, respectively, whereas the LVOT diameter measured by TTE was: 22 ± 1 mm. The ratio of AP to LR diameters measured by CMR was 0.87 ± 0.08 (median: 0.86; range: 0.78 - 0.94) and overall 74% of patients had a ratio < 0.9, thus confirming that most patients have an oval-shape of LVOT. There was no difference in AP to LR diameters ratio between bicuspid vs. tricuspid valves (0.83 ± 0.07 vs. 0.88 ± 0.08, p = NS)

**Table 2:** Comparison of Transthoracic Doppler-echocardiography (TTE) and Cardiovascular Magnetic Resonance (CMR) data

	TTE	CMR	
	Mean ± SD	Mean ± SD	p-value
Heart rate (bpm)	65 ± 12	66 ± 11	0.40
LVOT area (cm^2^)	3.84 ± 0.8	4.78 ± 1.05	< 0.001
AP/RL diameter ratio		0.87 ± 0.08	-
LVOT VTI (cm)	21 ± 4	15 ± 4	< 0.001
SV (mL)	80 ± 13	80 ± 18	0.68
Ao VTI (cm)	61 ± 22	57 ± 20	0.02
EOA (cm^2^)	1.53 ± 0.67	1.59 ± 0.73	0.17

LVOT: Left ventricular outflow tract; AP: Anterior-Posterior diameter; RL: Right-Left diameter; LVOT VTI: flow velocity time-integral in LVOT; SV: Stroke volume; Ao VTI: peak aortic velocity-time integral; EOA: valve effective orifice area.

### LVOT flow velocities and stroke volume

**Figure 4:**
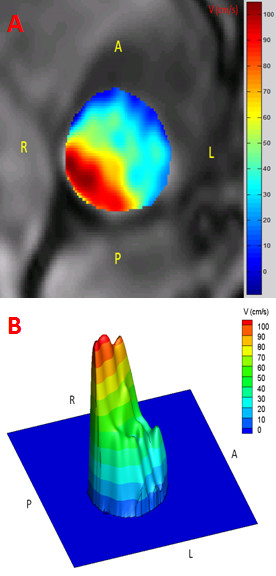
**Flow velocity profile obtained by CMR in the LV outflow tract (LVOT)**. The figure shows the flow velocity profile within the LVOT in a patient with AS. Panels A and B show the 2D and 3D flow velocity profile.

VTI_LVOT _measured by TTE was greater than that measured by CMR (bias = 14 cm and agreement limits: +1 to +26 cm) (Table 2). This may be due to the fact Doppler-echocardiography measures the flow velocity at the center of the LVOT, assuming an homogeneous and flat velocity profile, whereas CMR reveals that the flow velocity profile is skewed with greater velocities along the anterior and right aspects of the LVOT (Figure 4).

### Aortic valve EOA

**Figure 5:**
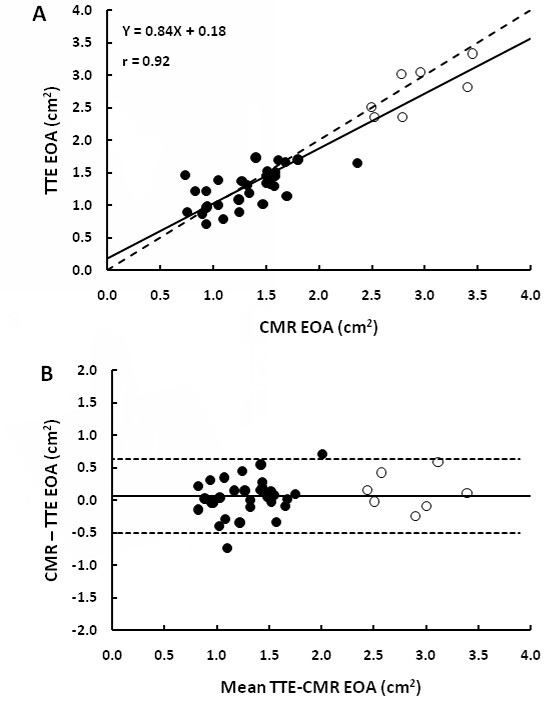
**Comparison of valve effective orifice area (EOA) measured by TTE versus by CMR**. Panel A shows the Pearson correlation plot. The solid line is the regression line and the dashed line is the identity line. Panel B shows the Bland-Altman plot. The solid line is the mean bias and dashed lines are ± 1.96 standard-deviations lines.

**Figure 6:**
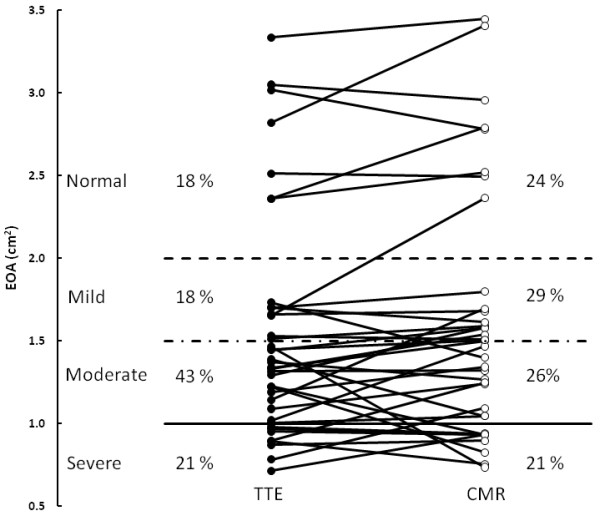
**Grading of aortic stenosis severity with the use of TTE- versus CMR-derived effective orifice areas (EOA)**.

Overall, there was a good correlation and concordance between EOA_TTE _and EOA_CMR _(1.53 ± 0.67 cm^2 ^vs. 1.59 ± 0.73 cm^2^, r = 0.92, bias = +0.06 cm^2^, agreement limits: -0.50 to +0.62 cm^2^; Figure 5). Nonetheless, 12 (39%) patients had a change in AS severity class when using the EOA_CMR _rather than the EOA_TTE _(Figure 6). Four (13%) patients were re-classified in a more severe class and 8 (26%) in a less severe class. Two (6%) patients with severe AS on the basis of EOA_TTE _were re-classified as moderate by EOA_CMR _and three (9%) patients with moderate AS on the basis of EOA_TTE _were classified as severe by EOA_CMR_.

### Measurement variability

In the subset of 15 subjects (11 AS patients and 4 control subjects) in whom the analyses of EOA were repeated on the same set of images, the intra- and inter- observer variability of EOA_TTE _was 5 ± 5% and 9 ± 5%, respectively, compared to 2 ± 1% and 7 ± 5% for CMR. In the subset of 5 patients in whom both the acquisition and analysis of images were repeated, the intra- and inter- observer variability of EOA_TTE _was 10 ± 8% and 12 ± 5%, respectively, compared to 8 ± 8% and 9 ± 8%, for EOA_CMR_.

## Discussion

Doppler-echocardiography is the method used to assess AS severity and guide therapeutic management in current practice. The valve EOA is one of the most frequently used Doppler-echocardiographic index to quantify stenosis severity. However, the measurement of valve EOA may not be feasible in a significant proportion of patients due to poor acoustic window and/or subvalvular flow acceleration. Moreover, given that the calculation of EOA requires the inclusion of 3 measures (LVOT diameter, LVOT VTI, and aortic jet VTI) in the continuity equation, this method may yield to relatively large measurement errors. Finally, there are often discordances between EOA and other Doppler-echocardiographic indices of stenosis severity, such as peak aortic jet velocity and transvalvular gradients. These discordances are particularly frequent in presence of low flow state conditions, where the gradients may be low despite the presence of a severe stenosis [18,19]. Discordant or inconclusive Doppler-echocardiographic findings may raise some uncertainty about the actual severity of the stenosis and therefore about the therapeutic management of the patient. There is thus an important need for additional non-invasive and accurate methods to corroborate stenosis severity in patients for whom Doppler-echocardiography does not provide a definitive conclusion with regard to AS severity.

Multidetector computed tomography is a powerful imaging modality to measure dimensions, surfaces and volumes of cardiac chambers. However, this method does not allow measurement of flow velocity and thereby does not permit the determination of valve EOA. CMR is a non-invasive, radiation-free imaging modality that allows quantification of flow velocity in the LVOT and aorta. Moreover CMR has superior temporal resolution compared to computed tomography.

The main finding of this study is that there is a good agreement between CMR and Doppler-echocardiography for the estimation of valve EOA. This study also confirms the results of previous studies that reported that Doppler-echocardiography underestimates the LVOT cross-sectional area compared to computed tomography imaging [20-26]. This overestimation is essentially related to the fact that Doppler-echocardiography assumes a circular LVOT shape, whereas, in fact, it is oval in most patients. However, as opposed to what was previously believed, this underestimation of LVOT area does not necessarily translate into underestimation of LV stroke volume and valve EOA. Indeed, TTE overestimates the LVOT VTI compared to CMR, which thus compensates the underestimation of LVOT area and yields to concordant estimates of valve EOA. With TTE method, it is assumed that: i) the flow velocity profile in the LVOT is flat, i.e. mean velocity equals peak velocity, and ii) the flow velocity profile is homogenous, i.e. measurement of velocity with the pulsed-wave Doppler sample volume positioned in the center of the LVOT accurately reflects the average velocity throughout the whole LVOT cross-section. However, as illustrated in Figure 4, CMR reveals that flow velocity profile is not flat and is often skewed with higher velocities along the anterior and right aspects of the LVOT. Hence, tracing of the contour of the peak velocity envelopes obtained by pulsed-wave Doppler at the center of the LVOT overestimates the actual mean velocity and the VTI in the LVOT. The overestimation of VTI_LVOT _by TTE somewhat counterbalances the underestimation of A_LVOT_. And consequently, the average stroke volume and EOA determined by TTE are similar to those determined by CMR.

### Aortic valve EOA

Several previous CMR studies have focused on the measurement of the area of the aortic valve orifice by planimetry [5,10-13]. However, it is important to underline that this "anatomic" orifice area (AOA) is not equivalent to the EOA. The latter indeed reflects the cross-sectional area of the vena contracta of the transvalvular flow jet [14,15]. The EOA is generally smaller than the AVA because there is a contraction of the flow downstream of the valve orifice. From a physiological standpoint, the transvalvular pressure gradient and thus the LV workload are essentially determined by the EOA and the magnitude of flow rate. The ratio EOA/AOA, i.e. the contraction coefficient, may vary from 0.6 to 1.0 depending on the shape of the valve inflow and the geometry of the valve orifice [14,15,27]. Hence, the EOA is superior to the AOA to accurately quantify the LV hemodynamic burden associated with the stenosis. Doppler-echocardiography and CMR are the two sole methods capable of measuring the valve EOA.

Our results are consistent with those of Caruthers et al., who reported a very good correlation between EOA determined by CMR with the use of continuity equation and that obtained by TTE (r = 0.83, SEE = 0.22 cm^2^) [6]. In a study where the stroke volume entered in the continuity equation was estimated by the Simpson method (i.e. LV end-diastolic volume minus LV end-systolic volume) instead of stroke volume measured in the LVOT, Yap et al. obtained an excellent correlation with TTE (r = 0.91, SEE = 0.17 cm^2^) [7]. Hagui et al. also proposed a hybrid method using the stroke volume measured by CMR and the aortic jet VTI obtained by TTE in the continuity equation [9]. This hybrid CMR-TTE method had a good agreement with the standard TTE method (bias = -0.01 cm^2^, limits of agreement: -0.36 to 0.34). The correlation between CMR- and TTE- derived EOAs reported in the present study appears to be better than those reported in previous studies. This may be due, at least in part, to differences in the population samples. Moreover, in the present study, we tested several locations for the measurement of the aortic jet VTI and found that highest velocities were obtained at 6 mm downstream to the valve orifice. These findings suggest that the vena contracta may actually be closer to the valve orifice compared to what was assumed (10 mm) in the previous studies [6-10].

In the present study, we also assessed the intra- and inter- observed variability: first, by repeating the EOA measurements with the use of the same sets of CMR and TTE images, and second, by repeating both acquisition and analysis of images. In both situations, CMR was found to have much less measurement variability compared to TTE, which lends further support to the reliability of this alternative imaging modality to confirm stenosis severity in the AS population.

## Clinical implications

Estimation of EOA by CMR should be contemplated when Doppler-echocardiographic measurement of EOA is not feasible or when the findings are discordant: e.g. valve EOA in the severe range (< 1.0 cm^2^) but mean transvalvular gradient in the moderate range (< 40 mmHg) or vice versa [18,19]. Recent studies have revealed that these discordances are frequent [3,28]. The first situation (small EOA and low gradient) is often found in presence of low transvalvular flow. The stroke volume and thus the transvalvular flow may indeed be significantly reduced not only in patients with low LV ejection fraction but also in those with preserved LVEF. This latter entity was recently described by our group and was termed: "paradoxical" low flow AS [3,29]. This entity is characterized by pronounced LV concentric remodelling, small LV cavity with impaired LV filling and reduced stroke volume despite preserved LVEF. These patients with paradoxical low flow AS, who represent approximately 15-20% of AS population, often exhibit discordance between EOA and gradient and accurate determination of stroke volume and EOA is crucial in these patients. CMR may be particularly useful in these patients to corroborate stenosis severity and guide therapeutic management.

## Limitations

The main limitations of this study are the relatively small number of patients with severe AS and the absence of a gold standard reference method. Unfortunately there is no such method available for in vivo measurement of valve EOA. The determination of valve EOA by catheterization with the use of the Gorlin formula also has important limitations and cannot be considered as a gold standard reference method [30]. Furthermore, this method is associated with increased risk of cerebral embolism [4].

## Conclusions

Underestimation of A_LVOT _by TTE is compensated by overestimation of VTI_LVOT_, thereby resulting in a good concordance between TTE and CMR for estimation of aortic valve EOA. CMR provides a non-invasive and reliable alternative to Doppler-echocardiography for the quantification of AS severity.

## Competing interests

The authors declare that they have no competing interests.

## Authors' contributions

All authors contributed to the scope and outline of the manuscript. JG wrote the final draft. All authors read and approved the final manuscript.
